# The catheter rendezvous technique for safe wire removal during Najuta prothesis implantation

**DOI:** 10.1016/j.jvscit.2025.102102

**Published:** 2025-12-15

**Authors:** Amir Arnautovic, Artis Knapsis, Sofie Lückerath, Waseem Garabet, Hubert Schelzig, Markus Udo Wagenhäuser

**Affiliations:** Clinic of Vascular and Endovascular Surgery, Medical Faculty and University Hospital Duesseldorf, Heinrich-Heine-University, Duesseldorf, Germany

**Keywords:** Aortic arch prothesis, Fenestrated prothesis, Endovascular, Penetrating aortic ulcer

## Abstract

Fenestrated aortic arch prostheses represent a robust therapeutic option for select pathologies. The Najuta is a semicustomized stent graft designed to achieve reliable sealing zones for the supra-aortic vessels through a variable number of fenestrations. A defining aspect of the procedure is the creation of a through-and-through wire (TATW), which is executed through a transaxillary approach via the fenestration of the brachiocephalic trunk. The omission of the catheter rendezvous technique for safe removal of the TATW, as described herein, may result in severe complications, including damage to the fabric or secondary dislocation of the stent graft, potentially leading to catastrophic outcomes such as aortic dissection or stroke. During implantation, the Najuta prosthesis is introduced using a 4-m soft Terumo wire configured in a transaxillary-transfemoral TATW arrangement. A loop is then formed around the aortic valve, facilitating the safe advancement of the prosthesis into the aortic arch. This loop configuration must be untied after implantation. If this is performed carelessly, excessive tension may be exerted on the medial edge of the brachiocephalic trunk fenestration, which may potentially lead to fabric damage or secondary dislocation of the entire prosthesis. To avoid this, catheters are inserted via both the transaxillary and transfemoral access points. These catheters meet at the loop area in a controlled “rendezvous” maneuver, enabling the safe removal of the TATW via the transaxillary access. The catheter rendezvous technique ensures the safe extraction of the loop TATW during the implantation of the Najuta prosthesis, thereby enhancing procedural safety and minimizing patient risk.

Endovascular treatment options for various aortic pathologies have advanced significantly in recent years. The aortic arch, however, remains a particularly challenging segment to address. A range of stent graft solutions are available for the endovascular treatment of this region, including fenestrated and branched stent grafts.^1^ The reported overall technical success rates for endovascular procedures in the aortic arch are highly encouraging.^1^

The Najuta thoracic stent graft system (SB-Kawasumi Laboratories, Inc) offers an effective solution for treating aortic arch pathologies requiring a landing in either zone 0 or 1, and it can even be applied to bovine aortic arches.^2^ This fenestrated stent graft system is a semicustomized device that does not necessitate reconstruction of the supra-aortic branches, thanks to its strategically placed fenestrations^3^^,^^4^ (Fig 1).

**Fig 1:**
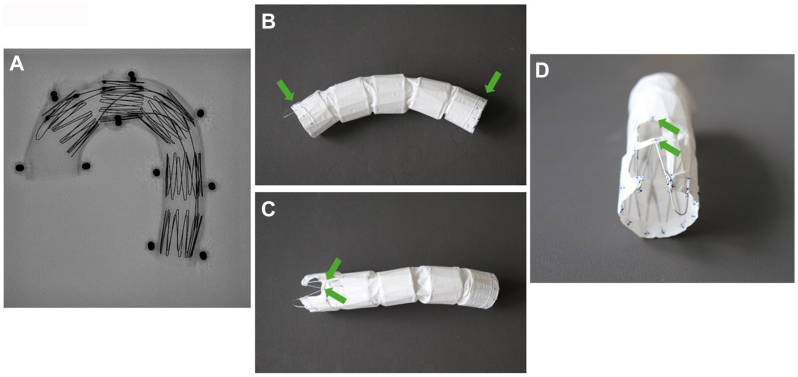
**(A)**, Radiographic (X ray) image of the Najuta stent graft, featuring self-expanding stainless-steel Z-stents. Each Z-stent measures 25 mm in length and is configured with dual struts along the greater curvature to enhance structural stability. The stainless-steel framework is precontoured to conform precisely to the anatomical curvature of the aortic arch. The stent graft is shown deployed within an aortic arch model. Lateral **(B)**, superior **(C)**, and anterior **(D)** views of the Najuta stent graft. The PTFE graft is affixed to the external surface of the stents exclusively at the proximal and distal extremities, as well as around the fenestrations (indicated by *green arrows*). The number of fenestrations (up to 3) within the PTFE fabric is variable and can be tailored to accommodate individual anatomical requirements. Note: The depicted Najuta stent graft differs between the images. *PTFE*, Polytetrafluoroethylene.

The insertion, positioning, and deployment of a Najuta stent graft require a through-and-through guidewire (TATW) technique using a 0.035-inch, 400-cm hydrophilic nitinol guidewire (Radifocus Guidewire M Non-Vascular; Terumo Corporation) with access via the right brachial/axillary artery and the femoral artery. The procedure involves advancing the endograft along the TATW while maintaining controlled tension from both the cranial and distal access points. Once the endograft tip enters the aortic arch, the tension is released, creating a loop of the TATW in the aortic root. This loop facilitates the precise advancement of the stent graft tip to its designated deployment position in the ascending aorta.^5^

In this context, we present a technique that enhances the safety of the implantation procedure, thereby improving both patient safety and clinical outcomes.

## Technique

The procedure is performed as part of the implantation of the semicustom fenestrated Najuta aortic arch prosthesis (Kawasumi Laboratories, Inc). Initially, a right transaxillary and transfemoral approach is established. After navigation of a 0.035-inch angled-tip glide wire (Terumo) into the descending thoracic aorta, it is exchanged for a 0.035-inch Lunderquist extra-stiff wire (Cook Medical) via a catheter. A 45-cm, 10F sheath (Cook Medical) is then advanced through the right transaxillary access to the origin of the brachiocephalic trunk (BCT). Subsequently, the Lunderquist extra-stiff wire is replaced with a 0.035-inch, 400-cm Nitinol hydrophilic guidewire, and a TATW is established via the transfemoral access using a 6F snare catheter (Merit Medical).

Next, another 0.035-inch angled-tip glide wire is introduced via the 10F sheath adjacent to the TATW and advanced into the ascending aorta. A pigtail catheter (Merit Medical) is placed over this wire to enable angiographic imaging of the aortic arch and supra-aortic branches, ensuring precise deployment of the Najuta prosthesis.

**Fig 2:**
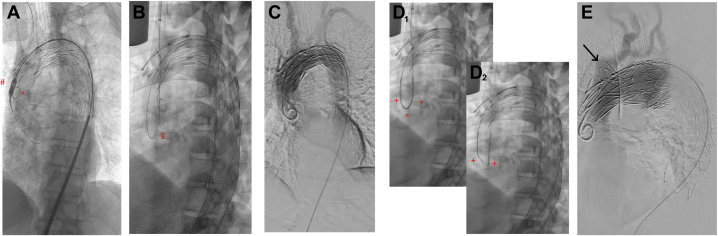
The catheter rendezvous technique for safe wire removal during Najuta stent graft implantation. **(A)**, Fully deployed Najuta stent graft. The tip of the delivery system (#) remains outside the sheath. A pigtail catheter (∗) is positioned aside the through-and-through wire (TATW) via the transaxillary access into the ascending aorta, allowing for angiography. **(B)**, Fully deployed Najuta stent graft. A loop of the TATW is now visible over the aortic valve (§), exiting through the transaxillary access and passing through the fenestration of the brachiocephalic trunk (BCT) in the stent graft. **(C)**, Final angiography in axial projection of the aortic arch demonstrating unobstructed perfusion of all supra-aortic branches. **(D_1_)**, Two vertebral catheters (+) are advanced toward the apex of the TATW loop (ˆ) via both transfemoral and transaxillary accesses. **(D_2_)**, The TATW can now be safely withdrawn while preserving the loop, minimizing the risk of secondary dislocation of the Najuta stent graft. This is achieved via either access route under carefully controlled, continuous traction and refeeding. Finally, the tips of the support catheters diverge, thus ending their “rendezvous,” with the TATW being localized in only one of the two catheters, allowing for its safe removal. **(E)**, Potential complication resulting from improper TATW removal based on local experience. Excessive tension may be transmitted to the wire at the BCT fenestration, potentially leading to damage to the stent graft fabric and dislocation of the endoskeleton (*black arrow*).

The Najuta prosthesis is then prepared for implantation and flushed using a pressure system to ensure complete deflation of the application system. After insertion of a 22-24F sheath via the transfemoral access, the prosthesis is advanced to the aortic arch and deflated again. A loop is created over the aortic valve with the TATW and deposited in position. The prosthesis is then advanced into the aortic arch, and after equalizing the angulation, angiography is performed to identify the supra-aortic branches. For deployment, arterial blood pressure is reduced to systolic values of <100 mm Hg. Once the target pressure is reached, the Najuta prosthesis is aligned with the markings on the supra-aortic branches according to the preoperative plan, and the first two rows of stents are deployed. After confirming free perfusion into the supra-aortic branches, the prosthesis is fully released (Fig 2, *A*). The tip of the delivery system is then returned to the sheath, while the TATW is continuously advanced to maintain the loop on the aortic valve, and the delivery system is retrieved as a whole.

On removal of the delivery system, the TATW loop on the aortic valve is guided out of the transaxillary access through the fenestration of the BCT from the Najuta prosthesis (Fig 2, *B*). After the final angiogram to verify the free perfusion of all supra-aortic branches (Fig 2, *C*), the TATW is safely removed, ensuring that the loop deposited on the aortic valve is preserved. This marks the final step of the complex endovascular procedure.

To enhance patient safety and prevent secondary dislocation of the endoskeleton or damage to the stent graft fabric, a catheter rendezvous maneuver can be used to securely retrieve the TATW. In this technique, two vertebral catheters are advanced via the transaxillary and transfemoral approaches. The catheters meet at the apex of the loop (Fig 2, *D*_*1*_), allowing the TATW to be safely removed through continuous traction via the transaxillary approach while refeeding it via the transfemoral approach. As traction is applied, the catheters “dance” to a point where they lose connection, thus concluding the “rendezvous” (Fig 2, *D*_*2*_). In this procedure, the two catheters act as protective sheaths for the TATW, preventing it from damaging the vascular wall or the stent graft fabric, particularly at the medial edge of the BCT fenestration.

This catheter rendezvous technique effectively prevents damage to the access vessel and/or the Najuta stent graft fabric, as well as mitigating the risk of secondary dislocation resulting from disproportionate tension. After the TATW is removed, the vertebral catheters are individually withdrawn after advancing a 0.035-inch angled-tip glide wire.

**Fig 3:**
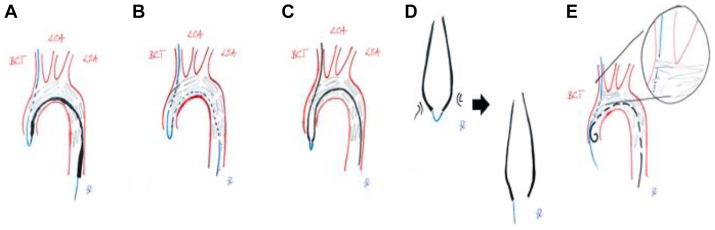
Schematic illustration of the catheter rendezvous technique for safe looped through-and-through wire (TATW) removal during Najuta stent graft implantation. **(A)**, The initial postdeployment scenario of the Najuta stent graft is depicted. The tip of the delivery system remains in place, whereas the TATW traverses the brachiocephalic trunk (BCT) and its corresponding fenestration, forming a loop at the level of the aortic valve. **(B)**, The situation after retrieval of the delivery system via the transfemoral approach is shown. The TATW remains positioned over the BCT and through its fenestration, with the loop still maintained at the aortic valve. **(C)**, A vertebral catheter is advanced simultaneously via both the transaxillary and transfemoral routes. The catheter tips are guided toward the loop of the TATW, establishing a connection through the wire and thereby facilitating alignment between the two access points. **(D)**, The TATW is then safely extracted by applying gentle, continuous traction from the transaxillary approach, accompanied by simultaneous refeeding from the transfemoral side. This coordinated movement of the catheters ensures a controlled and secure withdrawal. The procedure concludes when the catheters disengage from one another, signifying the completion of the “rendezvous.” **(E)**, Illustration of a potential complication from improper TATW removal: damage to the Najuta stent graft fabric at the medial edge of the BCT fenestration, caused by localized stress during transaxial tension in the absence of concurrent refeeding from the transfemoral route. The catheter rendezvous technique may effectively mitigate this risk by distributing tension evenly and maintaining graft integrity throughout the wire removal process.

It is important to note that the local center experience suggests that improper removal of the TATW poses a risk of secondary dislocation or uprighting of the Najuta prosthesis due to the tension exerted on the BCT fenestration. Such tension can not only compromise the fabric of the Najuta stent graft but may also lead to displacement of the endoskeleton (Fig 2, *E*). In alignment with the steps described above, the procedure is further visualized using schematic illustrations (Fig 3). Based on this experience, the catheter rendezvous technique has been established as a standard procedure to prevent such procedure-related complications with significant morbidity potential.

## Discussion

We herein present a potential technique for the safe removal of the transaxillary-transfemoral TATW after the placement of the Najuta stent graft for aortic arch pathologies, with the goal of preventing secondary dislocation and/or damage to the graft fabric.

Endovascular therapies have made significant advancements over the past decade. However, endovascular repair of the aortic arch carries a considerable risk of procedure-related complications, including stroke, spinal cord ischemia, and mortality.^6^ Fenestrated aortic arch stent grafts have emerged as a potential solution, addressing a wide range of aortic arch pathologies. Tsilimparis et al^7^ provided a comprehensive overview of the indications for such stent grafts, with postdissection false lumen aneurysm being the most common indication. Although the study reported valuable outcomes, including reintervention rates, the majority of procedures used Ishimaru zone 1 for the proximal landing. In contrast, the Najuta stent graft is specifically designed for landing in Ishimaru zone 0, making stent graft-specific outcomes particularly relevant and of significant interest.

Because the Najuta stent graft may contribute to preventing or reducing stent graft-related complications with potentially significant clinical consequences, the proposed catheter rendezvous technique could contribute to improving the described outcomes. However, the potential benefit in patient outcomes is yet to be demonstrated in long-term studies. That said, limited data on outcomes after Najuta implantation are available in the current literature.

In this regard, the Najuta stent graft has demonstrated its capability to achieve high technical and clinical success rates in an Italian registry of 76 patients, with 30-day follow-up success rates of 97.4% and 94.7%, respectively.^8^ Longer-term follow-up data are also available. In a larger cohort study between 2007 and 2013, the authors reported a freedom from aneurysm-related death of 97%, whereas overall survival was 67% at 3-year follow-up.^9^ Notably, after 7 years of follow-up, the freedom from aorta-related events might drop to 50.7%.^10^ Further, Sato et al^10^ also observed a type Ia endoleak (EL) rate of up to 27.8% over a period of 10 years. Others made similar observations, although the type Ia EL rates are somewhat lower. Notably, two retrospective analyses of a small patient cohort at a median follow-up of 2.5 years and a mean follow-up of 22.3 months described the type Ia EL rate at approximately 10%.^9^^,^^11^

In addition to the possible patient outcomes after Najuta stent graft placement, knowledge of possible bailouts strategies during the implantation procedure is crucial for the surgeon. The latest review of technical bailouts strategies provides potential solutions for removing the application system from the aortic arch.^5^ Also, other severe procedure-related complications such as a complete endograft collapse have been described recently.^12^

We now present here a further possibility to increase the safety of implantation by introducing a safe TATW removal option after the deployment of the Najuta stent graft. To remove the TATW, two surgeons must work together: one pulling on the wire via the transaxillary access while the second surgeon refeeds it from the lower transfemoral access. Failure to work in a coordinated manner can result in a pulling force on the TATW, which removes the loop and stretches the TATW. This tension can then lead to secondary stent graft dislocation and damage to the fabric because the TATW passes through the ostium of the BCT at this time. By using the catheter rendezvous technique, such risks are minimized, contributing to overall patient safety.

## Conclusions

The endovascular treatment options of the aortic arch for various pathologies are constantly growing. The catheter rendezvous technique presents an additional approach, which may contribute to enhancing procedural safety by introducing a technique for safe TATW removal after the deployment of the Najuta stent graft, which may protect against secondary stent graft dislocation and fabric damage.

## Funding

None.

## Disclosures

None.
